# Association between Post-Diagnosis Particulate Matter Exposure among 5-Year Cancer Survivors and Cardiovascular Disease Risk in Three Metropolitan Areas from South Korea

**DOI:** 10.3390/ijerph17082841

**Published:** 2020-04-21

**Authors:** Seulggie Choi, Kyae Hyung Kim, Kyuwoong Kim, Jooyoung Chang, Sung Min Kim, Seong Rae Kim, Yoosun Cho, Gyeongsil Lee, Joung Sik Son, Sang Min Park

**Affiliations:** 1 Department of Biomedical Sciences, Seoul National University Graduate School, Seoul 03080, Korea; seulggie@gmail.com (S.C.); kwkim238@gmail.com (K.K.); joomyjoo@gmail.com (J.C.); ksm9904@naver.com (S.M.K.); 2 Department of Family Medicine, Seoul National University Hospital, Seoul 03080, Korea; kyaehyungkim@gmail.com (K.H.K.); misslonghorn46@gmail.com (Y.C.); gespino.1gs@gmail.com (G.L.); medical114@naver.com (J.S.S.); 3 Institute for Public Health and Medical Service, Seoul National University Hospital, Seoul 03080, Korea; 4 College of Medicine, Seoul National University Hospital, Seoul 03080, Korea; sungkim20@snu.ac.kr

**Keywords:** cardiovascular disease, particulate matter, cancer survivor, metropolitan area

## Abstract

Cancer survivors are at an increased risk for cardiovascular disease (CVD). However, the association between particulate matter (PM) and CVD risk among cancer survivors (alive >5 years since diagnosis) is unclear. We investigated the risk of CVD among 40,899 cancer survivors within the Korean National Health Insurance Service database. Exposure to PM was determined by assessing yearly average PM levels obtained from the Air Korea database from 2008 to 2011. PMs with sizes <2.5 (PM2.5), <10 (PM10), or 2.5–10 (PM2.5–10) μm in diameter were compared, with each PM level exposure further divided into quintiles. Patients were followed up from January 2012 to date of CVD event, death, or December 2017, whichever came earliest. Adjusted hazard ratios (aHRs) and 95% confidence intervals (CIs) for CVD were calculated using Cox proportional hazards regression by PM exposure levels. Compared with cancer survivors in the lowest quintile of PM2.5 exposure, those within the highest quintile had a greater risk for CVD (aHR 1.31, 95% CI 1.07–1.59). Conversely, increasing PM10 and PM2.5–10 levels were not associated with increased CVD risk (*p* for trend 0.078 and 0.361, respectively). Cancer survivors who reduce PM2.5 exposure may benefit from lower risk of developing CVD.

## 1. Introduction

With the increasing incidence and the improved survival of patients with cancer, the number of cancer survivors is rapidly increasing in Korea. In 2016, 229,180 Koreans were newly diagnosed with cancer, while 78,194 died from cancer. The recent 5-year relative survival rate for cancer patients in Korea was 70.6%, which was similar to that reported in the US of 67.1% [1,2]. Nearly a million people in Korea have survived for more than 5 years after their cancer diagnosis, compared to over 11 million in the US [3,4]. Mortality from primary cancers accounts for up to 38.0% of all-cause mortality among cancer patients, while cardiovascular diseases (CVD) accounts for 11.3% [5]. Cardiovascular risk among these patients may be a consequence of the cardiotoxicity of treatment with anthracyclines, HER-2-targeted agents, and tyrosine kinase inhibitors, biological mechanisms such as inflammation and oxidative stress, and shared lifestyle risk factors for both cancer and CVD [6]. The increased risk for CVD has been shown among various cancers, including cancers of the breast, lung, prostate, pancreatic, hematologic, esophageal, kidney, and ovary [7,8,9].

Particulate matter (PM), defined as material suspended in the air in the form of minute solid particles or liquid droplets, is related to both cancer and CVD. It is currently classified as a Group 1 carcinogen by the International Agency for Research on Cancer (IARC) [10,11]. Moreover, PM has a dose–response relationship with carcinogenesis in various cancer types [10,11,12]. PM is also associated with subclinical atherosclerosis [13] and increased morbidity and mortality from CVD [14,15]. The size of PM has an influence on the pathophysiology of relative diseases and is generally classified into PM10 (<10 µm diameter), which is further subdivided into PM2.5–10 (2.5–10 µm), PM2.5 (<2.5 µm), and ultrafine (<0.1 µm) [16]. PM less than 10 µm can form deposits in airways and lungs, while the finest particles can reach the alveoli, enter the bloodstream, and cause systemic inflammation. While the harmful effects of PM among people with preexisting diseases such as CVD, respiratory diseases, and diabetes is well known [17], there is scarce evidence among cancer survivors. 

Exposure to PM affects short-term survival of patients with lung and liver cancers [18,19], as well as long-term survival of patients with breast cancer [20]. Although former investigators speculated that PM affects cancer progression and leads to poor outcomes, cardiovascular morbidity and mortality due to PM has not yet been studied among cancer survivors.

Using data from the Korean National Health Insurance Service (NHIS), we assessed the effects of PM concentration and diameter on the incidence of CVD among patients with 5-year survival following cancer diagnosis.

## 2. Experimental Section

### 2.1. Study Population

The Korean NHIS provides mandatory health insurance for all citizens [21], covering various health services including outpatient and inpatient department visits, pharmaceutical prescriptions, surgical procedures, and procedural and laboratory examinations. All citizens aged ≥40 years are eligible for a biannual health screening exam that includes a self-reported questionnaire on their past medical history, lifestyle behaviors, anthropometric measurements including height and weight, blood pressure, and blood and urine laboratory examinations [22]. The NHIS provides some of the data from these services for research purposes, and numerous epidemiological studies have used the data previously. Its validity is described in detail elsewhere [21,22,23]. 

“Health care utilization database” of NHIS includes the type of diseases, detailed treatment, and prescription of hospital inpatient department. [22] The ICD-10 code for cancer (C00–C96) and treatment modality such as chemotherapy was included in it. The health care utilization database included the whole population of over 50 million in South Korea. The ICD-10 code for cancer is registered by the doctor, and the patient is co-paid 95% by the government. The “Health screening database” was derived from the biannual health screening exam. It is smaller, and the participation of screening is around 40%–70% according to screening items. We have merged health screening and health care utilization databases.

A total of 46,334 patients in 2011 residing in three metropolitan areas in South Korea and newly diagnosed with cancer in 2006, survived until 2011. We excluded 544 patients who were aged <20 years at diagnosis, 2252 patients who had missing values for covariates, and 2639 patients who were diagnosed with CVD prior to the index date from further analyses. The final study population consisted of 40,899 cancer patients who had survived for more than 5 years after initial diagnosis. Starting from the index date of 1 January 2012, all subjects were followed up until the date of a CVD event, death, or 31 December 2017, whichever came earliest.

### 2.2. Ethical Considerations

This study was approved by the Seoul National University Institutional Review Board (IRB number: E-1905-148-1035). The requirement for informed consent was waived by the review board as the NHIS database is anonymized with use of its data guided by strict confidentiality guidelines.

### 2.3. Key Variables

Information on PM concentration and size was obtained from the Air Korea database which includes data on yearly average PM2.5 and PM10 levels for the administrative district areas in South Korea. The Air Korea database collects daily PM levels using more than 300 atmospheric monitoring sites distributed across the entire country. PM2.5 levels were available for three metropolitan cities (Seoul, Busan, and Incheon) starting from 2008, allowing us to determine the average PM level exposure from 2008–2011 for people residing in these three metropolitan cities which contain 50 administrative districts and reside over a third of the entire population of South Korea. Among the 50 districts of the cites under analysis, a total of 64 monitoring stations were located. While most districts had one monitoring station, for the 14 districts that had two monitoring stations, we used the average value for PM level exposure. The area of the districts ranged from 2.8 to 755.0 km squared. The mean (SD) area for the districts was 55.1 (79.9) km squared. PM2.5–10 levels were calculated by subtracting the PM10 levels from the PM2.5 levels. The average PM2.5, PM10, and PM2.5–10 levels were linked with the residential district codes for each participant, after which the participants were divided into quintiles of PM levels. 

Cancer survivors were defined as participants newly diagnosed with cancer in 2006 who survived until 2011 (at least 5 years). Cancer was defined as having a cancer diagnosis code (ICD-10: C00–C99) and the critical condition code for cancer as was defined in a previous study [24]. The critical condition code is applied when the patient is confirmed to be a cancer patient, after which the patient benefits from significant reimbursements for cancer-related treatment and management costs. Cardiotoxic chemotherapy was extracted from the inpatient hospital prescription database. For all patients, it was determined whether they were given cardiotoxic chemotherapy agents after cancer diagnosis and prior to the index date. Cardiotoxic chemotherapy agents included 5-fluorouracil, capcitabine, paclitaxel, cisplatin, trastuzumab, pertuzumab, bevacizumab, imatinib, dasatinib, nilotinib, ponatinib, vemurafenib, sorafenib, sunitinib, pazopanib, vandetanib, crizotinib, lapatinib, and erlotinib. The primary outcome, CVD, was defined as any hospitalization for coronary heart disease (CHD) or stroke for ≥2 days. Upon admission, the attending physicians are required to record a primary diagnosis for each patient according to the International Classification of Diseases, Tenth Revision codes. The ICD-10 codes for CHD (I20–I25) and stroke (I60–I69) were derived from the American Heart Association guidelines [25].

We considered the following covariates in the multivariate analysis: age (continuous, years), sex (categorical: men and women), household income (categorical: 1st, 2nd, 3rd, and 4th quintiles), area of residence (categorical: Seoul, Busan, and Incheon), and Charlson comorbidity index (continuous). Household income was derived from the insurance premium. The algorithm for calculating Charlson comorbidity index values from health claims data was adopted from a previous study [26].

### 2.4. Statistical Analysis

Differences in the distribution of covariates according to quintiles of PM2.5 or PM10 levels were determined using the Chi-squared test for categorical variables and the analysis of variance for continuous variables. The adjusted hazard ratios (aHRs) and 95% confidence intervals (CIs) for CVD, CHD, and stroke risk were calculated using the multivariate Cox proportional hazards regression. Kaplan–Meier analyses were conducted to assess the association of quintiles of PM2.5 with CVD, CHD, and stroke. Subjects within the lowest levels of PM2.5, PM10, and PM2.5–10 were used as the reference group. The association of PM2.5 levels with CVD risk was determined according to the cancer types, which included lung, breast, liver, stomach, colorectal, kidney, bladder, prostate, and smoking-related and obesity-related cancers. Smoking-related cancers included malignancies from the head and neck, esophagus, stomach, colorectum, liver, pancreas larynx, trachea, bronchus and lung, bladder, kidney, and acute myeloid leukemia [27]. Obesity-related cancers included breast, cervical, endometrial, uterine, colon, liver, gallbladder, pancreas, kidney, and esophageal cancers [28].

Statistical significance was determined as a *p*-Value <0.05 in a two-sided test. All data collection and statistical analyses were conducted using SAS Enterprise Guide 7.1 (SAS Institute, Cary, NC, USA).

## 3. Results

### 3.1. Patient Characteristics

Table 1 shows the characteristics of the study population. The number of subjects residing in the 1st, 2nd, 3rd, 4th, and 5th quintiles of PM2.5 levels were 8206, 8100, 7837, 9087, and 7669, respectively. The range of PM2.5 levels in order of increasing quintile groups were 19.8–23.9, 24.1–25.3, 25.4–26.4, 26.5–27.8, and 28.2–33.8 μg/m^3^. Participants residing in areas with the highest PM2.5 levels were younger, had higher household income, and had less comorbidities (all *p* < 0.001). The number of participants within the 1st, 2nd, 3rd, 4th, and 5th quintiles of PM10 levels were 8891, 7210, 7984, 8519, and 8295, respectively. The range of PM10 levels in order of increasing quintile groups were 35.5–49.1, 49.7–50.4, 50.4–52.6, 52.7–54.1, and 54.4–61.9 μg/m^3^. Compared to those residing in the lowest levels of PM10, cancer survivors residing in the highest levels of PM10 were younger, had higher household income, and had less comorbidities (all *p* < 0.001).

The mean follow-up duration was 5.53 ± 1.31 years, and the distribution of cancer according to cancer site is as follows: 636 subjects for lip, oral, and pharynx (C00–C14), 14,418 subjects for digestive organs (C15–C26), 1351 subjects for respiratory and intrathoracic organs (C30–C39), 188 subjects for bone and articular cartilage (C40–C41), 503 subjects for skin (C43–C44), 239 subjects for connective and soft tissue (C45–C49), 11,168 subjects for breast and female genital organs (C50–C58), 1271 subjects for male genital organs (C60–C63), 2138 subjects for urinary organs (C64–C68), 515 subjects for eye, brain, and central nervous system (C69–C72), 7017 subjects for endocrine glands and related structure (C73–C75), 109 subjects for secondary and ill-defined (C76–C80), and 1346 subjects for lymphoid, hematopoietic, and related tissue (C81–C96).

### 3.2. Association of PM Levels with CVD Risk

The association of PM2.5 levels with CVD risk is shown in Table 2. Compared to participants within the lowest quintile of PM2.5, those in the highest PM2.5 quintile group were at higher risk for CVD (aHR 1.31, 95% CI 1.07–1.59). Similarly, participants residing in the highest PM2.5 areas were at higher risk for CHD (aHR 1.47, 95% CI 1.10–1.95) compared to those within the lowest quintile. Moreover, there was a risk-increasing trend for CVD and CHD (*p* for trend 0.011) according to higher groups of PM2.5 quintiles. The risk for CVD according to PM10 quintile levels is shown in Table 3. Kaplan–Meier curves demonstrated significantly shorter CVD and CHD survival for subjects exposed to the highest quintile of PM2.5 in Figure S1.

Compared to those within the lowest quintile of PM10, cancer survivors with the highest exposure to PM10 did not have a higher risk for CVD (aHR 1.04, 95% CI 0.89–1.21), CHD (aHR 1.09, 95% CI 0.87–1.36), or stroke (aHR 0.99, 95% CI 0.80–1.22). Table 4 shows the association of PM2.5–10 levels with CVD risk among cancer survivors. Compared to subjects within the lowest quintile of PM2.5–10, participants residing in the highest quintile of PM2.5–10 did not have a higher risk for CVD (aHR 1.04, 95% CI 0.90–1.20), CHD (aHR 1.02, 95% CI 0.83–1.25), and stroke (aHR 1.06, 95% CI 0.87–1.29). Finally, the risk of CVD among survivors from lung, breast, liver, stomach, colorectal, kidney, bladder, prostate, smoking-related, and obesity-related cancers according to PM2.5 levels is shown in Table 5. There was a tendency towards increased CVD risk for higher PM2.5 levels, although the results were not significant due to the reduced number of participants and statistical power. 

Table S1 shows a stratified analysis of the association of PM2.5 with CVD risk according to subgroups of sex. The risk-increasing association of PM2.5 on CVD did not appear to differ according to sex (*p* for interaction > 0.05), although the results appeared to be stronger among women. 

Table S2 describes health behaviors, BMI, systolic BP, and serum glucose and total cholesterol concentration according to PM2.5 quintiles. A relatively small number (18,918 among 40,899) of subjects participated in laboratory tests and health behavior questionnaires. We conducted a sensitivity analysis (Table S3) among those who underwent health examinations during 2010–2011 (18,918 subjects) by additionally adjusting for lifestyle behaviors and results from laboratory exams, which are all biomarkers for future CVD risk. After additional adjustments for smoking, alcohol intake, physical activity, body mass index, systolic blood pressure, fasting serum glucose, and total cholesterol, the risk for CVD was higher among those residing in areas with high PM2.5 level.

## 4. Discussion

In the three metropolitan areas with over 40,000 cancer survivors, long-term exposure to PM was associated with increased CVD incidence. To our knowledge, this is the first study to identify an increasing effect of PM2.5 levels on CVD in long-term cancer survivors. The higher the concentration of PM2.5, the higher the risk was for CVD, confirming a concentration–responsive relationship. The hazard ratio for CVD increased from PM2.5 concentration of 28.2 µg/m^3^. 

In previous studies, PM2.5 has been reported to be the most pathogenic for CVD in the general population, with PM2.5–10 and PM10 levels having inconsistent results [16,29,30]. Our study also showed similar results. We found that a yearly average concentration of 28.2 µg/m^3^, which is above the yearly PM2.5 regulation guideline in South Korea (15 µg/m^3^), US (12 µg/m^3^), European Union (25 µg/m^3^), and Japan (15 µg/m^3^) [31], was associated with increased risk for CVD. 

In the general population, an increase in the PM2.5 concentration by 10 μg/m^3^ was associated with an increase in the number of hospital admissions for coronary artery disease, arrhythmias, heart failure, cerebrovascular disease, and peripheral artery disease [32]. Susceptible populations to poor health outcomes due to PM included children, older adults, those that were obese, had low socioeconomic status (SES), and specific genetic factors [33]. Unlike traditional Framingham risk factors, women were more susceptible to CVD when exposed to PM2.5 than men, possibly due to their smaller coronary arteries and microvessels [34,35,36]. 

In addition to these risk factors, cancer survivors may be more susceptible to the harmful effects of PM. They are often asymptomatic but have several times higher prevalence of CVD compared with the Framingham Heart Study population [37]. Chemotherapy agents for cancer patients have several vascular complications including endothelial dysfunction, capillary rarefaction, vascular remodeling, oxidative stress, platelet activation, thrombosis, and vasospasm [38]. In addition, radiotherapy impairs diastolic and systolic function [39]. The time-lapse between chemotherapy and radiotherapy and incident CVD can be long, lasting more than 10 years [40,41]. In the present study of long-term survivors, the risk-elevating association of PM2.5 with CVD was maintained after adjustments for cardiotoxic chemotherapy. Another factor, such as blood pressure rather than previous cardiotoxic chemotherapy, which has a significant association with PM concentration in our sensitivity analysis, can be addressed as a cause of increased CVD. Further research on multiple biomarkers should be performed. 

In the present study, breast and bladder cancer survivors were the most affected by PM2.5, which is consistent with a previous study [20]. PM seems to have a greater influence on CVD-susceptible cancers of all kinds. In the previous study, endometrial, breast, melanoma, prostate, and urinary bladder cancer were susceptible among cancers in 28 sites [5]. Breast cancer survivors treated with taxanes, vascular endothelial growth factor (VEGF) inhibitor, and hormonal agents experience increased thrombotic events, severe hypertension, and heart failure [38,39]. Moreover, there are the possible impact of the lifestyle paradox that women have more time- and energy-consuming lifestyle than men [42]. This corresponds to our further analysis that female cancer survivors are more vulnerable to PM. Future studies on the role of gender differences on the association of PM with CVD are needed.

The relationship between bladder cancer and PM remains controversial. Environmental and socioeconomic factors are known to affect bladder cancer mortality [43]. Those engaged in occupations such as taxi driver, truck driver, or street vendor are highly exposed to air pollution and have been reported to be at increased risk for bladder cancers [44]. However, there was no association reported between PM2.5 concentrations and bladder cancer incidence in 15 European cohort studies and one Spanish study [45,46]. The relationship between non-cancer mortality and PM has scarce evidence for bladder cancers as patients with bladder cancers had high to no risk for CVD in the previous studies [5,8]. In our long-term bladder cancer survivors, the concentration of 24.1–25.3 and 28.2–33.8 µg/m^3^ of PM2.5 was associated with CVD. Further research in other large-scale populations is needed. 

In the present study, there was no association between PM concentration and stroke in cancer survivors. Both hemorrhagic and ischemic stroke risk were increased in cancer patients [47,48] even after 10 years of surviving [49] in previous studies. This phenomenon may need to be investigated further. 

In our study, participants living at high concentrations of PM2.5 were younger than those who did not and were more likely to have a Charlson comorbidity index of less than 3. Since participants in the city are expected to commute to work, they may be younger compared to the suburban participants. In our data, there was a lack of information on occupation or the geographic location of the workplace, and thus should be addressed in future studies.

Our study has several limitations. First, cancer stage was not considered in this study. The severity of primary cancer may have altered the susceptibility of the particulate matter. The Korean National Health Insurance Service (NHIS) data, which was used in this study, is not a cancer registry. On the other hand, the Korea National Cancer Incidence Database (KNCIDB) is a hospital-based cancer registry system that has the stages of cancer. However, the overall cancer prevalence rates using the two data are quite similar in the previous studies, and this study suggests that NHIS data are a feasible data source for cancer survivorship [50,51]. Second, operationally defined CVD may be incorrectly coded. Unstable angina is one of the most up-coded conditions [52], and there is also the possibility that patients with multiple CVD could be down-coded should the doctor not have recorded all ICD codes for multiple complex conditions. However, the operational diagnosis was validated and had been widely used in previous studies [53,54]. Third, ischemic and hemorrhagic strokes could not be differentiated due to lack of data. Fourth, the PM monitoring station is less dense in some suburban districts. The area of the districts ranged from 2.8 to 755.0 km squared. Moreover, the study population was from three metropolitan cities, so the results may not be generalizable to rural areas. Fifth, the residential areas and areas where the population spends most of their time (such as the workplace) may be different. Sixth, the individual CVD risk factors (smoking, diet, alcohol use, access to care, obesity, blood pressure, lipid profile, and family history) were not identified in our main analysis, although non-smokers had a greater risk for air-pollution-related disease than current smokers in a previous study [12]. However, after additional adjustments for smoking, alcohol intake, physical activity, body mass index, systolic blood pressure, fasting serum glucose, and total cholesterol, the risk for CVD was higher among those residing in areas with high PM2.5 level (Table S3). We have included the population without laboratory tests to ensure sufficient numbers for each cancer type stratification.

## 5. Conclusions

We found that higher levels of PM2.5 were associated with increased CVD risk among 5-year cancer survivors, implying that cancer survivors are a vulnerable group when exposed to high PM levels. Among the various cancer types, breast and bladder cancer survivors were the most susceptible to the CVD risk–elevating effects of PM. The strengths of this study include a large sample of the Korean population with good quality control. Future studies will be needed to evaluate the impact of PM exposure reduction among cancer survivors

## Figures and Tables

**Table 1:** Descriptive characteristics of the study population.

Variables	Particulate Matter, Quintiles					*p*-Value
	1st (Lowest)	2nd	3rd	4th	5th (Highest)	
PM2.5						
Number of participants	8206	8100	7837	9087	7669	
Range, μg/m^3^	19.8–23.9	24.1–25.3	25.4–26.4	26.5–27.8	28.2–33.8	
Age, years, mean (SD)	59.1 (13.0)	59.8 (13.3)	59.4 (13.2)	59.5 (12.9)	57.9 (13.2)	<0.001
Sex, *N* (%)						
Men	3094 (37.7)	2945 (36.4)	2988 (38.1)	3589 (39.5)	2862 (37.3)	<0.001
Women	5112 (62.3)	5155 (63.6)	4849 (61.9)	5498 (60.5)	4807 (62.7)	
Income, quartiles, *N* (%)						
1st (highest)	3821 (46.6)	3643 (45.0)	4148 (52.9)	4206 (46.3)	3064 (40.0)	<0.001
2nd	1669 (20.3)	1616 (20.0)	1391 (17.8)	1861 (20.5)	1780 (23.2)	
3rd	1091 (13.3)	1110 (13.7)	986 (12.6)	1201 (13.2)	1184 (15.4)	
4th (lowest)	1625 (19.8)	1731 (21.4)	1312 (16.7)	1819 (20.0)	1641 (21.4)	
Charlson comorbidity index, *N* (%)						
1	1711 (21.0)	1609 (19.9)	1508 (19.2)	1815 (20.0)	1714 (22.4)	<0.001
2	2676 (32.6)	2547 (31.4)	2684 (34.3)	2858 (31.5)	2470 (32.2)	
≥3	3808 (46.4)	3944 (48.7)	3645 (46.5)	4414 (48.6)	3485 (45.4)	
PM10						
Number of participants	8891	7210	7984	8519	8295	
Range, μg/m^3^	35.5–49.1	49.7–50.4	50.4–52.6	52.7–54.1	54.4–61.9	
Age, years, mean (SD)	59.7 (13.1)	58.8 (13.0)	59.7 (13.1)	58.8 (13.2)	58.7 (13.2)	<0.001
Sex, *N* (%)						
Men	3344 (37.6)	2700 (37.5)	3016 (37.8)	3259 (38.3)	3159 (38.1)	0.824
Women	5547 (62.4)	4510 (62.6)	4968 (62.2)	5260 (61.7)	5136 (61.9)	
Income, quartiles, *N* (%)						
1st (highest)	3921 (44.1)	3575 (49.6)	3776 (47.3)	4129 (48.5)	3481 (42.0)	<0.001
2nd	1856 (20.9)	1337 (18.5)	1616 (20.2)	1674 (19.7)	1834 (22.1)	
3rd	1219 (13.7)	988 (13.7)	1054 (13.2)	1112 (13.1)	1199 (14.5)	
4th (lowest)	1895 (21.3)	1310 (18.2)	1538 (19.3)	1604 (18.8)	1781 (21.5)	
Charlson comorbidity index, *N* (%)						
1	1878 (21.1)	1407 (19.5)	1629 (20.4)	1648 (19.3)	1806 (21.8)	<0.001
2	2718 (30.6)	2448 (34.0)	2598 (32.5)	2857 (33.5)	2614 (31.5)	
≥3	4295 (48.3)	3355 (46.5)	3757 (47.1)	4014 (47.1)	3875 (46.7)	

Particulate matter levels determined by 4-year average levels during 2008–2011. *P*-value calculated by Chi-squared test for categorical variables and analysis of variance for continuous variables. Acronyms: PM, particulate matter; SD, standard deviation; N, number of participants. The Charlson comorbidity index was used to measure 17 comorbid disease statuses.

**Table 2:** Adjusted hazard ratios (95% CI) for cardiovascular disease according to post-diagnosis PM2.5 levels among 5-year cancer survivors.

Variables	PM2.5, Quintiles					*p* for Trend
	1st (lowest)	2nd	3rd	4th	5th (highest)	
Cardiovascular disease						
Events	359	329	325	349	365	
Person-years	45,317	44,937	43,626	50,065	42,207	
aHR (95% CI)	1.00 (reference)	1.02 (0.86–1.19)	1.06 (0.90–1.24)	1.11 (0.96–1.27)	1.31 (1.07–1.59)	0.011
Coronary heart disease						
Events	158	133	133	202	166	
Person-years	45,317	44,937	43,626	50,065	42,207	
aHR (95% CI)	1.00 (reference)	1.06 (0.83–1.36)	1.11 (0.86–1.42)	1.19 (0.96–1.46)	1.47 (1.10–1.95)	0.011
Stroke						
Events	201	196	192	237	199	
Person-years	45,317	44,937	43,626	50,065	42,207	
aHR (95% CI)	1.00 (reference)	0.98 (0.79–1.21)	1.02 (0.83–1.26)	1.06 (0.87–1.26)	1.18 (0.91–1.55)	0.257

Particulate matter levels determined by 4-year average levels during 2008–2011. Adjusted hazard ratio calculated by Cox proportional hazards regression analysis after adjustments for age, sex, household income, area of residence, Charlson comorbidity index, and cardiotoxic chemotherapy. Acronyms: PM, particulate matter; aHR, adjusted hazard ratio; CI, confidence interval.

**Table 3:** Adjusted hazard ratios (95% CI) for cardiovascular disease according to post-diagnosis PM10 levels among 5-year cancer survivors.

Variables	PM10, Quintiles					*p* for Trend
	1st (lowest)	2nd	3rd	4th	5th (highest)	
Cardiovascular disease						
Events	394	267	384	387	385	
Person-years	49,015	40,138	44,160	47,328	45,510	
aHR (95% CI)	1.00 (reference)	0.98 (0.83–1.15)	1.18 (1.02–1.36)	1.21 (1.05–1.41)	1.04 (0.89–1.21)	0.078
Coronary heart disease						
Events	181	105	162	157	185	
Person-years	49,015	40,138	44,160	47,328	45,510	
aHR (95% CI)	1.00 (reference)	0.88 (0.69–1.13)	1.14 (0.92–1.42)	1.18 (0.94–1.48)	1.09 (0.87–1.36)	0.111
Stroke						
Events	213	162	222	228	200	
Person-years	49,015	40,138	44,160	47,328	45,510	
aHR (95% CI)	1.00 (reference)	1.05 (0.85–1.30)	1.20 (0.99–1.46)	1.24 (1.02–1.51)	0.99 (0.80–1.22)	0.348

Particulate matter levels determined by 4-year average levels during 2008–2011. Adjusted hazard ratio calculated by Cox proportional hazards regression analysis after adjustments for age, sex, household income, area of residence, Charlson comorbidity index, and cardiotoxic chemotherapy. Acronyms: PM, particulate matter; aHR, adjusted hazard ratio; CI, confidence interval.

**Table 4:** Adjusted hazard ratios (95% CI) for cardiovascular disease according to post-diagnosis PM2.5–10 levels among 5-year cancer survivors.

Variables	PM2.5–10, Quintiles					*p* for Trend
	1st (Lowest)	2nd	3rd	4th	5th (Highest)	
Cardiovascular disease						
Events	419	326	286	423	363	
Person-years	46,989	43,996	42,215	50,648	42,304	
aHR (95% CI)	1.00 (reference)	1.03 (0.88–1.20)	0.94 (0.80–1.11)	1.10 (0.95–1.27)	1.04 (0.90–1.20)	0.361
Coronary heart disease						
Events	203	128	116	178	167	
Person-years	46,989	43,996	42,215	50,648	42,304	
aHR (95% CI)	1.00 (reference)	0.92 (0.72–1.17)	0.87 (0.68–1.11)	1.03 (0.82–1.28)	1.02 (0.83–1.25)	0.576
Stroke						
Events	216	198	170	245	196	
Person-years	46,989	43,996	42,215	50,648	42,304	
aHR (95% CI)	1.00 (reference)	1.11 (0.90–1.37)	1.00 (0.80–1.24)	1.16 (0.95–1.41)	1.06 (0.87–1.29)	0.468

Particulate matter levels determined by 4-year average levels during 2008–2011. PM2.5–10: particulate matter with sizes between 2.5 and 10 μm. Adjusted hazard ratio calculated by Cox proportional hazards regression analysis after adjustments for age, sex, household income, area of residence, Charlson comorbidity index, and cardiotoxic chemotherapy. Acronyms: PM, particulate matter; aHR, adjusted hazard ratio; CI, confidence interval.

**Table 5:** Adjusted hazard ratios (95% CI) for cardiovascular disease according to post-diagnosis PM2.5–10 levels among 5-year cancer survivors in various cancer types.

Variables	PM2.5, Quintiles					*p* for Trend
	1st (Lowest)	2nd	3rd	4th	5th (Highest)	
Lung cancer						
Events	12	9	10	9	12	
Person-years	841	799	846	946	783	
aHR (95% CI)	1.00 (reference)	0.71 (0.29–1.73)	0.83 (0.34–1.98)	0.79 (0.33–1.89)	2.46 (0.93–6.52)	0.389
Breast cancer						
Events	34	30	41	36	48	
Person-years	8013	8016	7250	8591	7461	
aHR (95% CI)	1.00 (reference)	1.08 (0.63–1.86)	1.60 (0.97–2.65)	1.04 (0.65–1.66)	2.25 (1.32–3.85)	0.045
Liver cancer						
Events	14	9	10	5	12	
Person-years	1006	962	996	1171	853	
aHR (95% CI)	1.00 (reference)	0.90 (0.35–2.31)	1.04 (0.41–2.63)	0.34 (0.12–0.96)	0.84 (0.23–2.98)	0.107
Stomach cancer						
Events	79	70	77	90	81	
Person-years	7618	7601	6931	8867	7624	
aHR (95% CI)	1.00 (reference)	0.96 (0.68–1.35)	1.14 (0.81–1.60)	0.99 (0.73–1.35)	1.33 (0.88–2.02)	0.379
Colorectal cancer						
Events	81	70	54	86	62	
Person-years	6160	6007	5630	6466	5588	
aHR (95% CI)	1.00 (reference)	0.88 (0.62–1.24)	0.77 (0.53–1.12)	1.00 (0.74–1.36)	1.17 (0.74–1.86)	0.630
Kidney cancer						
Events	13	6	6	15	11	
Person-years	1154	1079	1070	1182	947	
aHR (95% CI)	1.00 (reference)	0.50 (0.18–1.38)	0.55 (0.20–1.54)	1.07 (0.50–2.28)	1.65 (0.60–4.56)	0.301
Bladder cancer						
Events	13	23	13	29	19	
Person-years	1117	1062	1061	1518	966	
aHR (95% CI)	1.00 (reference)	2.86 (1.34–6.12)	1.62 (0.70–3.75)	1.77 (0.91–3.43)	2.44 (1.00–5.95)	0.210
Prostate cancer						
Events	20	17	21	28	15	
Person-years	1115	1073	1502	1276	737	
aHR (95% CI)	1.00 (reference)	0.86 (0.43–1.69)	0.83 (0.43–1.59)	1.26 (0.70–2.26)	1.23 (0.52–2.90)	0.304
Smoking-related cancer						
Events	226	201	185	253	218	
Person-years	19,067	18,508	17,502	21,442	17,977	
aHR (95% CI)	1.00 (reference)	0.99 (0.81–1.22)	0.99 (0.80–1.22)	1.00 (0.84–1.20)	1.34 (1.04–1.73)	0.201
Obesity-related cancer						
Events	166	137	133	174	161	
Person-years	21,160	21,073	18,987	22,623	19,772	
aHR (95% CI)	1.00 (reference)	0.88 (0.69–1.13)	0.97 (0.76–1.24)	0.98 (0.79–1.21)	1.33 (0.99–1.78)	0.209

Particulate matter levels determined by 4-year average levels during 2008–2011. Smoking-related cancer included head and neck, esophagus, stomach, colorectum, liver, pancreas larynx, trachea, bronchus and lung, bladder, kidney cancer, and acute myeloid leukemia. Obesity-related cancer included breast, cervical, endometrial, uterine, colon, liver, gallbladder, pancreas, kidney, and esophageal cancer. Adjusted hazard ratio calculated by Cox proportional hazards regression analysis after adjustments for age, sex, household income, area of residence, and Charlson comorbidity index. Acronyms: PM, particulate matter; aHR, adjusted hazard ratio; CI, confidence interval.

## Supplementary Materials

The following are available online at https://www.mdpi.com/1660-4601/17/8/2841/s1, Figure S1: Kaplan–Meier survival analysis of (**A**) cardiovascular disease, (**B**) coronary heart disease, and (**C**) stroke survival according to quintiles of PM2.5 concentration, Table S1: Stratified analysis on the association of post-diagnosis PM2.5 levels among 5-year cancer survivors with cardiovascular disease according to subgroups of sex. Table S2 Descriptive characteristics of the study population that underwent health screening examinations. **Table S3**. Hazard ratios for cardiovascular disease according to post-diagnosis PM2.5 levels among 5-year cancer survivors who underwent health screening examinations.
