# Supplementary material for: Association between Post-Diagnosis Particulate Matter Exposure among 5-Year Cancer Survivors and Cardiovascular Disease Risk in Three Metropolitan Areas from South Korea
Source: Int J Environ Res Public Health. 2020 Apr 21;17(8):2841. doi: 10.3390/ijerph17082841 (PMC7215375; doi:10.3390/ijerph17082841)
Supplement: Supplementary file 1 [file ijerph-17-02841-s001.pdf]

Supplementary Materials:

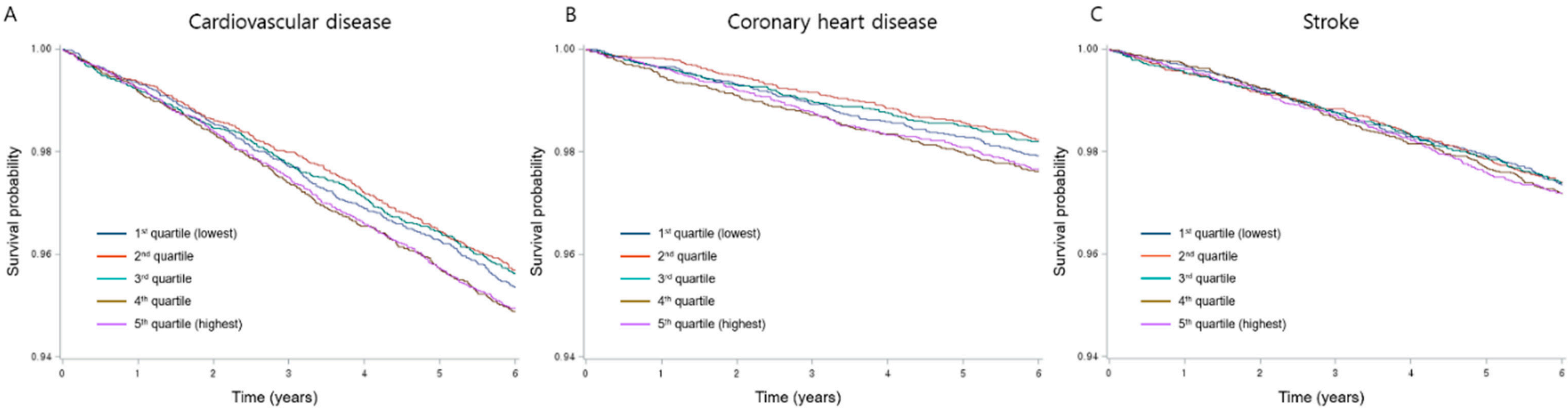

Figure S1. Kaplan–Meier survival analysis of (**A**) cardiovascular disease, (**B**) coronary heart disease, and (**C**) stroke survival according to quintiles of PM2.5 concentration.

Table S1. Stratified analysis on the association of post-diagnosis PM2.5 levels among 5-year cancer survivors with cardiovascular disease according to subgroups of sex.

	PM2.5, Quintiles					*p* for Trend
	1st (Lowest)	2nd	3rd	4th	5th (Highest)	
Men						
Cardiovascular disease						
Events	204	178	182	240	185	
Person-years	16,184	15,601	15,946	18,966	15,056	
aHR (95% CI)	1.00 (reference)	0.96 (0.77–1.19)	0.97 (0.78–1.20)	1.01 (0.84–1.22)	1.11 (0.84–1.46)	0.528
Coronary heart disease						
Events	95	77	84	116	92	
Person-years	16,184	15,601	15,946	18,966	15,056	
aHR (95% CI)	1.00 (reference)	0.96 (0.70–1.33)	1.03 (0.75–1.41)	1.08 (0.82–1.41)	1.33 (0.91–1.96)	0.189
Stroke						
Events	109	101	98	124	93	
Person-years	16,184	15,601	15,946	18,966	15,056	
aHR (95% CI)	1.00 (reference)	0.95 (0.71–1.26)	0.92 (0.69–1.23)	0.96 (0.74–1.24)	0.92 (0.62–1.37)	0.707
Women						
Cardiovascular disease						
Events	155	151	143	199	180	
Person-years	29,133	29,336	27,681	31,098	27,151	
aHR (95% CI)	1.00 (reference)	1.09 (0.85–1.39)	1.17 (0.92–1.49)	1.23 (0.99–1.51)	1.55 (1.17–2.05)	0.003
Coronary heart disease						
Events	63	56	49	86	74	
Person-years	29,133	29,336	27,681	31,098	27,151	
aHR (95% CI)	1.00 (reference)	1.21 (0.82–1.81)	1.21 (0.81–1.82)	1.35 (0.97–1.87)	1.64 (1.06–2.53)	0.021
Stroke						
Events	92	95	94	113	106	
Person-years	29,133	29,336	27,681	31,098	27,151	
aHR (95% CI)	1.00 (reference)	1.01 (0.75–1.38)	1.14 (0.84–1.54)	1.14 (0.87–1.51)	1.49 (1.03–2.15)	0.048

Particulate matter levels determined by 4-year average levels during 2008–2011. Adjusted hazard ratio calculated by Cox proportional hazards regression analysis after adjustments for age, sex, household income, area of residence, Charlson comorbidity index, and cardiotoxic chemotherapy. Acronyms: PM, particulate matter; aHR, adjusted hazard ratio; CI, confidence interval.

Table S2 Descriptive characteristics of the study population that underwent health screening examinations.

	PM2.5, Quintiles					
	**1st (Lowest)**	**2nd**	**3rd**	**4th**	**5th (Highest)**	***p* Value**
Smoking, *N* (%)						
Never smoker	2923 (74.6)	2825 (74.6)	2553 (75.5)	3094 (72.7)	2574 (72.1)	0.001
Past smoker	710 (18.1)	666 (17.6)	621 (18.4)	813 (19.1)	685 (19.2)	
Current smoker	285 (7.3)	296 (7.8)	209 (6.2)	352 (8.3)	312 (8.7)	
Alcohol intake, times per week, *N* (%)						
0	2906 (74.2)	2735 (72.2)	2468 (73.0)	3061 (71.9)	2623 (73.5)	0.067
0–1	481 (12.3)	484 (12.8)	421 (12.4)	537 (12.6)	455 (12.7)	
1–2	228 (5.8)	216 (5.7)	205 (6.1)	306 (7.2)	208 (5.8)	
3–4	199 (5.1)	228 (6.0)	184 (5.4)	204 (4.8)	175 (4.9)	
≥5	104 (2.7)	124 (3.3)	105 (3.1)	151 (3.6)	110 (3.1)	
Physical activity, times per week, *N* (%)						
0	1785 (45.6)	1675 (44.2)	1483 (43.8)	1872 (44.0)	1693 (47.4)	0.035
1–2	605 (15.4)	637 (16.8)	553 (16.4)	696 (16.3)	543 (15.2)	
3–4	580 (14.8)	536 (14.2)	520 (15.4)	618 (14.5)	501 (14.0)	
5–6	414 (10.6)	420 (11.1)	416 (12.3)	484 (11.4)	366 (10.3)	
7	534 (13.6)	519 (13.7)	411 (12.2)	589 (13.8)	468 (13.1)	
Body mass index, kg/m^2, mean (SD)	23.4 (3.1)	23.5 (3.1)	23.4 (3.1)	23.4 (3.1)	23.5 (3.2)	0.097
Systolic blood pressure, mmHg, mean (SD)	121.5 (15.4)	123.8 (15.3)	122.3 (15.5)	122.9 (15.4)	123.3 (16.1)	<0.001
Fasting serum glucose, mg/dL, mean (SD)	99.5 (21.0)	99.7 (21.1)	98.8 (21.2)	99.6 (22.6)	100.1 (23.3)	0.221
Total cholesterol, mg/dL, mean (SD)	193.9 (36.2)	193.2 (43.6)	193.4 (36.9)	193.2 (37.0)	192.9 (36.3)	0.848

Particulate matter levels determined by 4-year average levels during 2008–2011. *p*-value calculated by Chi-squared test for categorical variables and analysis of variance for continuous variables. Acronyms: PM, particulate matter; SD, standard deviation; N, number of participants.

Table S3. Hazard ratios for cardiovascular disease according to post-diagnosis PM2.5 levels among 5-year cancer survivors who underwent health screening examinations.

	PM2.5, Quintiles					*p* for Trend
	**1st (Lowest)**	**2nd**	**3rd**	**4th**	**5th (Highest)**	
Cardiovascular disease						
Events	155	139	142	195	171	
Person-years	22,307	21,614	19,233	23,992	20,118	
aHR (95% CI)	1.00 (reference)	1.03 (0.80–1.32)	1.20 (0.94–1.54)	1.13 (0.91–1.40)	1.37 (1.03–1.83)	0.037
Coronary heart disease						
Events	81	69	58	101	78	
Person-years	22,307	21,614	19,233	23,992	20,118	
aHR (95% CI)	1.00 (reference)	1.08 (0.76–1.54)	1.03 (0.71–1.48)	1.14 (0.85–1.53)	1.28 (0.85–1.94)	0.245
Stroke						
Events	74	70	84	94	93	
Person-years	22,307	21,614	19,233	23,992	20,118	
aHR (95% CI)	1.00 (reference)	0.98 (0.69–1.39)	1.37 (0.98–1.92)	1.12 (0.83–1.52)	1.47 (0.98–2.20)	0.071

Particulate matter levels determined by 4-year average levels during 2008–2011. Adjusted hazard ratio calculated by Cox proportional hazards regression analysis after adjustments for age, sex, household income, area of residence, Charlson comorbidity index, cardiotoxic chemotherapy, smoking, alcohol intake, physical activity, body mass index, systolic blood pressure, fasting serum glucose, and total cholesterol. Acronyms: PM, particulate matter; aHR, adjusted hazard ratio; CI, confidence interval.
